# Differences in Radiation Use in Left Atrial Appendage Occlusion Procedures Among Electrophysiology and Structural Operators

**DOI:** 10.1016/j.jscai.2024.102458

**Published:** 2025-01-21

**Authors:** Zachary Dean Demertzis, Ela Ahmad, Karan Chhabra, Nolan Shoukri, David Haines, Simon Dixon, Nishaki Mehta

**Affiliations:** a Department of Cardiovascular Disease, Corewell Health William Beaumont University Hospital, Royal Oak, Michigan; b Department of Internal Medicine, Corewell Health William Beaumont University Hospital, Royal Oak, Michigan; c Oakland University William Beaumont School of Medicine, Rochester, Michigan

**Keywords:** Left atrial appendage occlusion, radiation, electrophysiology, structural cardiology, radiation safety, atrial fibrillation

Atrial fibrillation is the most prevalent sustained cardiac arrhythmia, with its clinical impact predominantly attributable to risk of stroke^1^ emanating from a left atrial appendage thrombus.^2^ Long-term bleeding complications and considerations have led to the advent of alternative strategies, including implantation of left atrial appendage occlusion (LAAO) devices, that are noninferior to oral anticoagulation,^3^ thereby allowing patients to avoid such agents and their attendant bleeding consequences.

LAAO implantation is typically performed percutaneously employing fluoroscopy and echocardiography (transesophageal or intracardiac), with an average radiation dose of 6700 μGy⋅m^2^.^4^ Radiation exposure (RE) to operators and staff is still inextricably attendant in such procedures, as is the orthopedic burden of wearing personal protective lead aprons. The adverse occupational health consequences of performing fluoroscopy-based procedures have been the subject of much recent clinical scientific study and societal advocacy emphasizing the guiding principle of radiation safety of “as low as reasonably achievable” to reduce the associated adverse health effects (eg, premature cataracts, malignant tumors, and orthopedic injuries).^4^

LAAO implantations are performed by both structural cardiologists (SCs) and electrophysiologists (EPs). Societies representing SCs (Society for Cardiovascular Angiography & Interventions) and EPs (Heart Rhythm Society) have embraced advocacy efforts to enhance training to reduce RE complications and thereby mitigate occupational health hazards.^5^ The purpose of the present study was to compare operator (and patient) RE and use among EPs and SCs performing LAAO implantation.

We conducted a single-center, retrospective cross-sectional analysis across 80 months from April 2017 to November 2023 of all LAAO procedures. All patients who underwent an LAAO procedure had a CHA_2_DS_2_-VASc score ≥2 and were unable to tolerate anticoagulation due to associated bleeding. Cases involving concurrent procedures (eg, patent foramen ovale closure, transcatheter edge-to-edge repair, coronary angiography) during LAAO were excluded from the analysis.

We stratified procedural metrics, including procedure duration, fluoroscopy time, air kerma, and dose area product (DAP), by operator subspecialty. Adverse events were defined as procedural complications including a new pericardial effusion, perforation, or vascular injury. Peri-device leak was categorized as 0 to 3 mm, 3 to 5 mm, and >5 mm on 30-day postprocedural transesophageal echocardiogram. Primary operator years of experience was divided into quartiles (0-5 years, 5-10 years, 10-15 years, and >15 years). Continuous variables are described as means and categorical variables as frequency rates and percentages. The *t* test was used for continuous variables, and the *χ*^*2*^ test was used for categorical variables.

A total of 476 LAAO procedures were performed by EP (n = 277) and SC (n = 199) operators. A descriptive analysis of the study population concluded that the patient demographics (age, sex, and body mass index) were similar between both operator groups. Preprocedural imaging was statistically different between both groups, with EPs utilizing coronary computed tomography angiography (CCTA) (151 vs 80; *P* = .027) and SCs utilizing transesophageal echocardiogram (74 vs 89; *P* < .001) (Table 1). However, 33 months from the start of our review, CCTA was the predominant preprocedural imaging modality used by both groups (Supplemental Tables S1 and S2).

**Table 1:** Descriptive analytics, procedural characteristics, and complications of LAAO procedures for EP and SC operators

	EP (n = 277)	SC (n = 199)	*P*
Age, y	75	76	.763
Sex			NS
Male	171 (62%)	124 (62%)	
Female	106 (38%)	75 (38%)	
Body mass index, kg/m^2^	29.88	29.09	.161
Number of operators	4	6	
Operator experience, y			<.001
0-5	7 (3%)	32 (16%)	<.001
5-10	105 (38%)	42 (21%)	.001
10-15	29 (10%)	29 (15%)	.206
>15	136 (49%)	96 (48%)	.895
LAAO device			<.001
Watchman	224 (81%)	187 (94%)	.129
Amulet	53 (19%)	12 (6%)	<.001
Preprocedural imaging			<.001
None	52 (18.8%)	30 (15.1%)	.338
TEE	74 (26.7%)	89 (44.7%)	<.001
CCTA	151 (54.5%)	80 (40.2%)	.027
Procedure duration, min	60	62	.276
Fluoroscopy time, min	11.3	14.8	<.001
Air kerma, mGy	360.47	535.17	<.001
DAP, μGy⋅m^2^	5953.54	7720.31	<.005
Complications	6 (2.2%)	6 (2%)	.545
Pericardial effusion	4	2	.674
Perforation	0	1	.238
Vascular injury	0	1	.238
Other	2	2	.740
Peri-device leak	59 (21.3%)	61 (30.7%)	.113
0-3 mm	51	50	.117
3-5 mm	6	9	.153
>5 mm	2	2	.740

CCTA, coronary computed tomography angiography; DAP, dose area product; EP, electrophysiologist; LAAO, left atrial appendage occlusion; SC, structural cardiologist; TEE, transesophageal echocardiogram.

There was a significant difference in the number of cases performed by quartiles of years of experience (*P* < .001); however, the difference was predominantly among junior operators (7 vs 32, 0-5 years; *P* < .001; 105 vs 42, 5-10 years; *P* < .001). There were no differences in number of cases performed between the senior operators (>10 years) (Table 1).

Procedure duration between EP and SC operators showed no significant difference (60 vs 62 min, *P* = .276). However, EP operators had significantly shorter fluoroscopic times (11.3 vs 14.8 minutes, *P* < .001), lower air kerma (360.47 vs 535.17 mGy, *P* < .001), and lower DAP (5953.52 vs 7720.31 μGy⋅m^2^, *P* < .005) than SC operators. Independent predictors of higher DAP during LAAO procedures were operator specialty (SC: OR, 3.16; 95% CI, 2.03-5.01; *P* < .001), patient sex (male: OR, 7.5; 95% CI, 4.69-12.4; *P* < .001), and patient body mass index (OR, 1.17; 95% CI, 1.13-1.23; *P* < .001). Total complication (6 vs 6; *P* = .545) and peri-device leak (59 vs 61; *P* = .113) rates were similar between EP and SC operator groups (Table 1).

A subgroup analysis of radiation usage by operator experience showed no significant difference between specialties among junior operators (<10 years of experience); however, senior EP operators used less radiation with no difference in procedure duration compared to SC operators (Supplemental Table S3).

Our study revealed that the amount of radiation used in LAAO procedures was significantly less when performed by EP trained operators compared with SC trained operators. There was no statistically significant difference in procedure duration, peri-device leaks, or overall complication rates.

To our knowledge, this is the first study comparing radiation use between EP and SC operators. Our findings of lower radiation use among EP operators could be attributed to their reliance on alternative imaging modalities (ie, echocardiography) in navigating the procedure and use of CCTA for preprocedural planning. Other speculative factors may include the increased familiarity of EP operators with trans-septal access and catheter manipulation within the left atrium. Other factors could be that senior SC operators would have been “grandfathered” into performing structural procedures without formal training.

Our study’s limitations include its single-center, retrospective design with 10 total primary operators. Another limitation is the lack of standard fluoroscopic and cinematic frame rates (frames per second [fps]) across labs, although these are typically preset at 7.5 fps and 15 fps, respectively. A key limitation of the present study is the lack of measurement of RE per case operator; further studies employing online RE measurements of individual operators and staff will be necessary. Despite these limitations, the present study delineates an opportunity to achieve as low as reasonably achievable RE through hospital-wide standardization and trans-specialty collaboration exploiting the expertise singular to both specialties.

## Figures and Tables

**Supplementary figure 1:**
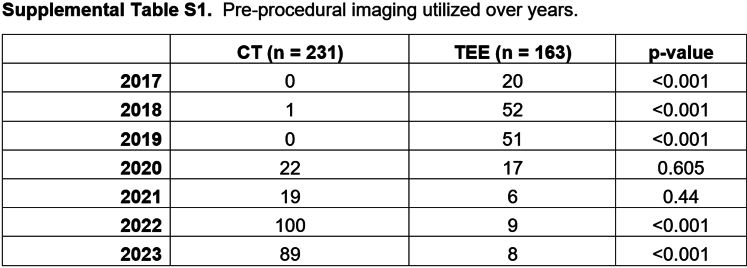


**Supplementary figure 2:**
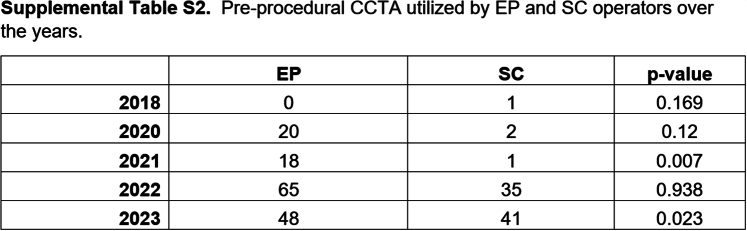


**Supplementary figure 3:**
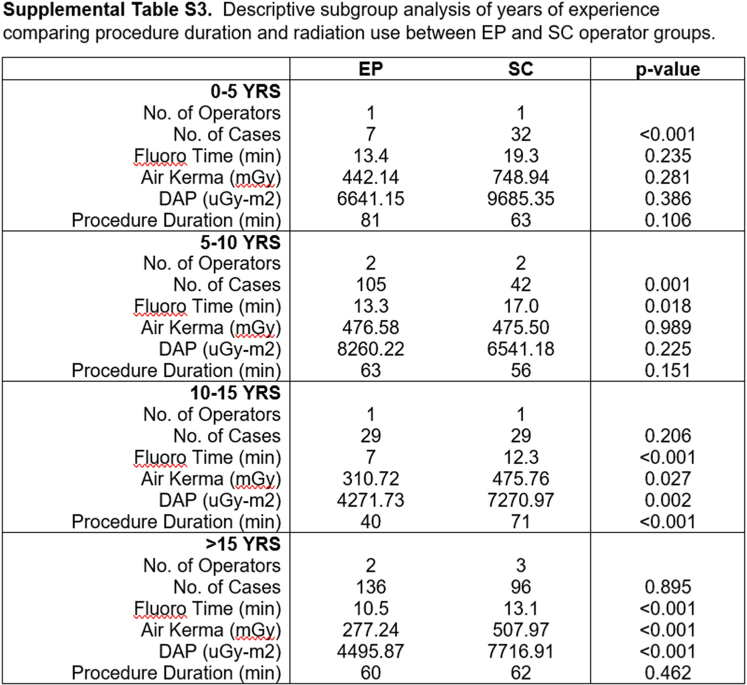


## Footnotes

To access the supplementary material accompanying this article, visit the online version of the *Journal of the Society for Cardiovascular Angiography & Interventions* at 10.1016/j.jscai.2024.102458.
